# Accelerometer‐based daily physical activity monitoring in patients with postpartum sacroiliac joint dysfunction: a case–control study

**DOI:** 10.1080/23335432.2024.2396277

**Published:** 2024-09-07

**Authors:** Sem M. M. Hermans, Jasper Most, Martijn G.M. Schotanus, Henk van Santbrink, Inez Curfs, Wouter L. W. van Hemert

**Affiliations:** a Department of Orthopaedic Surgery, Zuyderland Medical Center, Heerlen, the Netherlands; b Care and Public Health Research Institute (CAPHRI), Maastricht University, Maastricht, the Netherlands; c Department of Neurosurgery, Maastricht University Medical Center, Maastricht, the Netherlands; d Department of Neurosurgery, Zuyderland Medical Center, Heerlen, the Netherlands

**Keywords:** Sacroiliac joint, sacroiliac joint dysfunction, minimally invasive sacroiliac joint fusion, activity monitoring, spine

## Abstract

Patients with low back pain caused by sacroiliac joint (SIJ) dysfunction have an impaired quality of life, due to reported pain, disability and activity limitations. There is increasing evidence that minimally invasive sacroiliac joint fusion (MISJF) results in improvement in pain, disability and quality of life in these patients. Some studies have reported improvements in daily physical activity following MISJF but based on bias-prone self-reports. Our aim was to provide objective data on daily physical activity in patients with SIJ dysfunction. Daily physical activity in daily life of participants was measured using a triaxial accelerometer for seven consecutive days, before surgery and 3 months after surgery. Recorded daily activities were the daily number of events and total time spent sitting or lying, standing, walking, cycling, high-activity and number of steps and sit-to-stand transfers. The quality of life was assessed by the validated Dutch EQ-5D-5 L-questionnaire. No statistical differences were observed between daily physical activity in patients with SIJ dysfunction before and 3 months after MISJF. As compared to matched controls, high-intensity physical activity was lower in both the pre- and postoperative period (*p* = 0.007) for patients with SIJ dysfunction. The quality of life improved significantly in patients after MSIJF, from 0.418 to 0.797 (*p* = 0.021) but did not reach the level of controls (1.000). Daily physical activity in patients with postpartum SIJ dysfunction does not improve 3 months following MISJF, while quality of life does improve significantly. The discrepancy between these two observations is food for new research.

## Introduction

Sacroiliac joint (SIJ) dysfunction, a chronic musculoskeletal disease, leads to physical dysfunction and inactivity, negatively impacting quality of life (QoL) due to pain, disability, and activity limitations (Westerterp 2014). Daily physical activity is associated with improved physical and cognitive function, and reduces the risk of falls and even the risk of death (Powell et al. 2011; Warburton and Bredin 2017). To evaluate treatment efficacy for musculoskeletal diseases, it is important to monitor daily physical activity in individuals. Following other orthopedic treatments, e.g. hip and knee arthroplasties, daily physical activity improvement is surprisingly negligible, despite significant improvements in self-reported outcomes relating to daily physical activity (Elfving et al. 2007; Vitztum and Kelly 2015; Arnold et al. 2016; Almeida et al. 2018). This dissimilarity can potentially be explained by a large variability between patients, and by the fact that questionnaires are insensitive and prone to overestimation, due to subjectivity (Dunlop et al. 2011).

Current literature indicates that minimally invasive sacroiliac joint fusion (MISJF) is associated with improvements in pain, disability, and QoL for patients with SIJ dysfunction (Hermans et al. 2022; Chang et al. 2022). One can postulate that daily physical activity might also improve following MISJF. Studies on self-reported daily physical activity suggest improvements in daily physical activity (Darr et al. 2018; Martin et al. 2020), but objective data, of daily physical activity collected using gold-standard methodology in patients with SIJ dysfunction are unavailable.

In this case-controlled study, daily physical activity was measured in patients with postpartum SIJ dysfunction before and after MISJF and compared to age-, BMI-, gender- and postpartum-matched controls.

## Material & methods

### Participants

Between January 2021 and October 2021 10 patients scheduled for MISJF because of unilateral or bilateral SIJ dysfunction agreed to participate in this study. A case-controlled age and gender-matched healthy, postpartum control group (*n* = 11) was used for comparison (Table 1). All participants were informed about the study, and written informed consent was obtained prior to participation. This research has been approved by the IRB of the authors’ affiliated institution. Healthy participants were post-partum females aged 25 to 45 without history of SIJ dysfunction or other lower back related illness. All participants were asked to function according to their normal habits during daily physical activity monitoring.

**Table 1.** Characteristics of subjects.

	Patients (*N* = 8)	Controls (*N* = 11)	P-value
Age (years)	43.0 (33.5:46.3)	35.0 (33.0:36.5)	0.138
BMI (kg/m2)	25.4 (23.0:30.1)	23.1 (22.2:23.9)	0.09
Number of previous pregnancies 1 2 3 4	1 4 2 1	6 4 1 -	0.173
Years postpartum	5.5 (4.1:17.0)	4.7 (3.3:5.5)	0.258

All values are median with interquartile range (1:3). P-value refers to Mann – Whitney U test or Chi-Square test for categorical variable (N pregnancies).

### Outcome

For the patient group, daily physical activity tracking was performed between 12 and 1 week(s) before surgery and 3 months following surgery. Control patients were measured at least 6-month postpartum. Daily physical activity in the daily life of participants was measured using triaxial accelerometer (AM; GC Dataconcepts LLC, Waveland, USA) for seven consecutive days. The AM was attached onto the lateral side of the upper leg, left or right as preferred by the participant. Based on previously published principles, accelerometer data were processed and analysed using self-constructed algorithms for feature detection and activity classification written in Matlab (MATLAB R2010a, The MathWorks Inc., Natick, Massachusetts, USA) (Mathie et al. 2004; Preece et al. 2009; Verlaan et al. 2015). Activity parameters calculated were percentages of total time (duration) spent sitting or lying (inactive), standing, walking, cycling, high-activity (active) and number of steps and sit-to-stand transfers. The AM was only worn during waking hours with a minimum of 8 h a day and removed at night and during showering or other water activities. The quality of life was assessed by the validated Dutch EQ-5D-5 L-questionnaire (‘best health state’) and the EQ self-reported health status visual analogue scale (VAS) that records the respondent’s self-rated health (0–100, 100 being ‘best imaginable health state’) (Versteegh et al. 2016). EQ-5D-5 L-questionnaires were completed before the start of daily physical activity monitoring.

### Statistical analysis

Statistical analyses were carried out using IBM SPSS statistics 27. Descriptive data (means, SD, proportions) were generated for all variables. Statistically significant differences between both groups were analyzed with nonparametric Mann–Whitney U test, since the group sizes were small. To compare activity parameters between groups, Independent Samples Kruskal Wallis test was used. The chi-square test was used for categorical variables. A p-value ≤0.05 was considered to be statistically significant.

## Results

Eight patients completed this study. Two patients were excluded as the data were incomplete. The patient and control groups were statistically comparable regarding demographic characteristics (Table 1).

**Figure 1.**
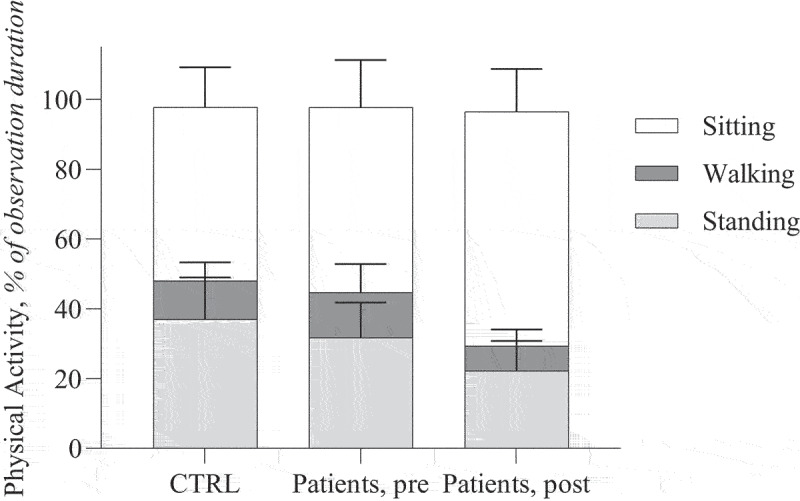
Average daily physical activity (of more than 1% of total daily physical activity) across groups.

When comparing the patient group to the control group, patients exhibited a significantly lower quality of life prior to surgery. However, daily total physical activity and low-to-medium intensity physical activity did not differ significantly between the two groups (Figure 1). Only high-intensity physical activity was notably reduced in patients before surgery.

*CTRL: controls*

Following surgery, quality of life improved in patients 3 months postoperatively compared to preoperatively, from 0.460 to 0.797 (*p* = 0.021). EQ-5D-5 L VAS also improved from 53 to 70 (*p* = 0.011). These postoperative scores did not reach comparable levels to that of healthy controls (Table 2).

**Table 2.** Quality of life results.

	Preoperative (1)	Postoperative (2)	Controls (3)	P-value
EQ-5D-5 L score	0.460 (0,291:0.516)	0.797 (0.691:0.818)	1.000 (1.000:1.000)	1-2: 0.021 1-3: < 0.001 2-3: < 0.001
EQ-5D-5 L VAS	53 (35:61)	70 (58:85)	90 (85:93)	1-2: 0.011 1-3: < 0.001 2-3: 0.007

All values are median with interquartile range (1:3). *P*-value refers to Mann – Whitney U test.

**Figure 2.**
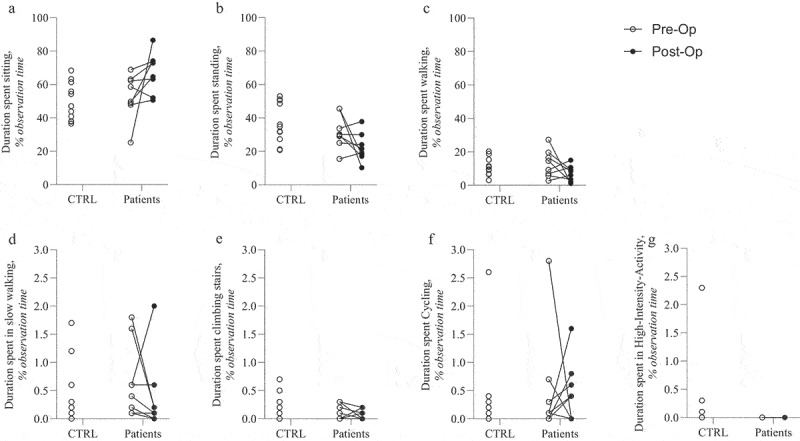
Detailed daily physical activity among individuals.

The percentage of each physical activity type contributing to total physical activity did not change significantly between pre- and post-operative groups (Figure 1). Activities accounting for less than 1% of total daily physical activity, such as slow walking, stair climbing, cycling, and high-intensity training (HIT), are not included in the figure. Additionally, daily physical activity varied greatly between individual patients (Figure 2).

In patients, the changes before and after surgery are depicted. CTRL: controls, Op: operative

## Discussion

This observational case-controlled cohort study showed that daily physical activity levels of patients with postpartum SIJ dysfunction did not increase 3 months after MISJF. This objective data also revealed that, during the study period, patients perform no high-intensity physical activity. Quality of life improved during the study period but did not reach level of healthy controls.

Currently, no literature exists on objective daily physical activity in patients with SIJ dysfunction. In a recent randomized trial, (Dengler et al. 2019) investigated several clinical outcomes of MISJF through questionnaires, including pain, walking distance, disability level and work status. All outcomes improved over the 24-month follow-up period. However, only pain, walking distance and disability level were already statistically significantly improved 3 months following MISJF. In contrast to work status, which was decreased 3 months postoperatively compared to preoperatively. Walking distance and work status further improved at 6-, 12- and 24-months follow-up, while pain and disability score remained stable at further follow-up moments. These data suggest that further functional improvement is expected beyond 3 months.

As QoL in patients with SIJ dysfunction is comparably low to that of patients with lumbar spinal conditions, comparing available objective data on daily physical activity might be insightful (Cher and Reckling 2015). A recent study by (Coronado et al. 2020) investigated daily physical activity in 53 patients who underwent lumbar spine surgery, 6 weeks, 3 and 6 months after surgery and found significant improvements over this time period. Another study by (Gilmore et al. 2019) stated that walking time in the first week after lumbar surgery is a predictor of significant improvement in function at 6 months postoperatively. In both studies, no preoperative comparison was available. In patients with osteoarthritis (e.g. hip and knee) requiring arthroplasty, pre- and postoperative data are available, and we know that daily physical activity does often not improve following surgery, while health-related QoL and social mobility do (Ethgen et al. 2004; Vogel et al. 2011; Stevens-Lapsley et al. 2011). These findings are in line with the findings of this study, as no statistically significant improvement in daily physical activity was observed in patients with SIJ dysfunction following MISJF, while EQ-5D-5 L score improved, however, not to the level of healthy controls. As enhanced QoL is a motivator for daily physical activity, one may expect that both improve (Gill et al. 2013). Potentially, the discrepancy between self-reported improvement and actual health-gain following surgery is overestimated by patients. Or perhaps the increase in QoL is a result of fewer pain complaints and not directly in enhanced daily physical activity.

In the current study group, mean daily physical activity parameters for patients did not differ pre- and postoperatively. This not only means that no improvement is observed, but to the contrary, no deterioration is observed either. This indicates that patients with SIJ dysfunction return to their preoperative daily physical activity level 3 months following surgery, thus recovered from surgery. These daily physical activity levels are no different from matched controls, except for high-intensity physical activity. The latter was the only parameter that differed between patients and matched controls. High-intensity physical activity comprises sport activities. The findings of this study therefore suggest that patients with postpartum SIJ dysfunction tend to avoid participating in sports. This observation prompts us to consider the intricate relationship between pain, physical activity, and lifestyle choices. It is plausible that pain and physical limitations play a significant role in this avoidance behavior, highlighting the profound impact of musculoskeletal conditions on daily functioning. Exploring participants’ engagement in sports activities before the onset of dysfunction could provide valuable insights into their current behavior.

### Strength & limitations

A strength of our study is the sensor-based measurements, which gives a more reliable representation of daily physical activity compared to previous studies with self-reported outcomes (Dengler et al. 2019). The small sample size is a limitation of the current study, and therefore, this study is not appropriate for responder and non-responder analyses. The small sample size is mainly caused by strict inclusion criteria and because this study serves as a pilot study. To reduce heterogeneity, only patients with SIJ dysfunction, as a cause of previous pregnancy, were included. SIJ dysfunction of post-partum origin is a prevalent cause of SIJ dysfunction, as noted in our previously published cohort study (Raj et al. 2021). Another limitation to the current study is the short follow-up period. Three months postoperatively might be too early to expect large improvements in daily physical activity. Patients are still in their rehabilitation process at this time point. Furthermore, in a large number of patients, bilateral SIJ complaints are present, for which a second surgery is needed, where the contralateral SIJ is fused to further alleviate complaints. In these patients, further improvement can still be expected following a second surgery. Figure 2 also indicates that changes in daily physical activity individually differed following MISJF. Therefore, it might be interesting to further investigate associations between patient characteristics (e.g. BMI, age, duration of complaints) and daily physical activity response to MISJF. Regardless of these limitations, we were able to detect differences in daily physical activity between patients with post-partum SIJ dysfunction and matched controls, in terms of high-intensity activity.

## Conclusion

This study demonstrates that daily physical activity of patients with SIJ dysfunction is comparable to that of healthy matched controls, except for high-intensity physical activity which is lower in patients. Preoperative QoL is significantly lower in patients compared with matched controls. Postoperative daily physical activity in patients does not improve, while QoL does improve significantly, however not to the level of healthy individuals.
